# Preparation and characterisation of ethambutol with β-cyclodextrin: a comprehensive molecular insight into host–guest interaction

**DOI:** 10.55730/1300-0527.3493

**Published:** 2022-08-10

**Authors:** Soen Qeng GOH, Qiao Hui CHAN, Rohana ADNAN, Nurul Yani RAHIM

**Affiliations:** School of Chemical Sciences, Universiti Sains Malaysia, Penang, Malaysia

**Keywords:** β-cyclodextrin, tuberculosis drug, ethambutol, inclusion complex

## Abstract

The increase in new cases of drug resistance to first-line drugs such as ethambutol (ETB) makes it necessary to develop improvements for antituberculosis drugs. A new method for improving the bioavailability of active pharmaceutical ingredient (API) was investigated for preventing drug resistance and side-effects of antituberculosis drugs. In this study, an antituberculosis drug delivery system using β-cyclodextrin (β-CD) as the supramolecular carrier of ETB was prepared using the kneading method. The inclusion behaviour of β-CD/ETB inclusion complex in solid and liquid state was investigated. The inclusion complex was characterized using Fourier transform infrared spectroscopy, thermogravimetric analysis, nuclear magnetic resonance (NMR) spectroscopy, and UV–visible spectroscopy. The ^1^H and nuclear Overhauser effect spectroscopy NMR results indicated the hydrophobic interaction between β-CD and ETB. Meanwhile, the Benesi-Hildebrand equation was used to calculate the formation constant (K) of β-CD/ETB complex in natural condition, pH4, and pH9, which were 105.43, 107.06, and 119.11, respectively. The stoichiometry ratio of β-CD/ETB complex was proven to be 1:1.

## 1. Introduction

**Figure 1:**
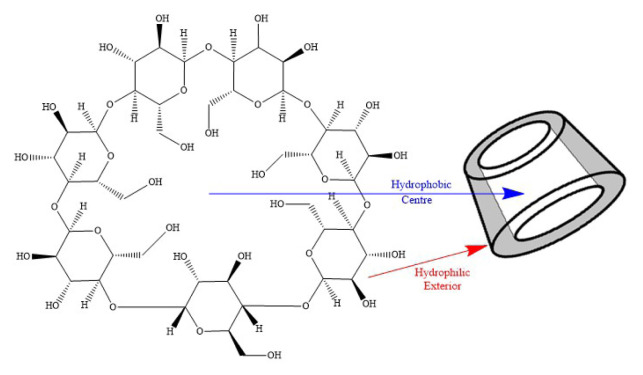
β-cyclodextrin.

Supramolecular chemistry is a discipline of chemistry that relies heavily on molecular recognition. It entails the host and guest forming certain bond orientation. Among all the prospective hosts, cyclodextrins (CDs) appear to be the most promising, as they can accommodate a wide range of guest molecules with the right polarity and size. CDs are oligosaccharides that are produced when starch undergoes enzymatic degradation, which contain six to eight D-(+)-glucopyranose monomers interconnected by α-1,4-linkages, in which they are named α-, β-, and γ-CD, respectively [1]. However, α-CD is regarded as unfitting for most drugs due to its small cavity space, and γ-CD is relatively costly which is not economical for mass production in the long run [2]. With that being said, the β-CD variant is one of the most abundant natural oligomers and possesses seven glucose units where the cavity is hydrophobic, and the exterior is strongly hydrophilic (Figure 1). This unique characteristic of β-CD enables the formation of various inclusion complexes with its large cavity to accommodate desired molecules via host-guest interaction. Various chemical interactions can be found in the inclusion complex, for instance hydrophobic, van der Waals, dipole-dipole, and hydrogen bonding interactions. Thus, the interaction can serve to modify the physical, chemical and biochemical properties of guest molecules, and the application of these guest molecules will ultimately be enhanced [3–6].

**Figure 2:**
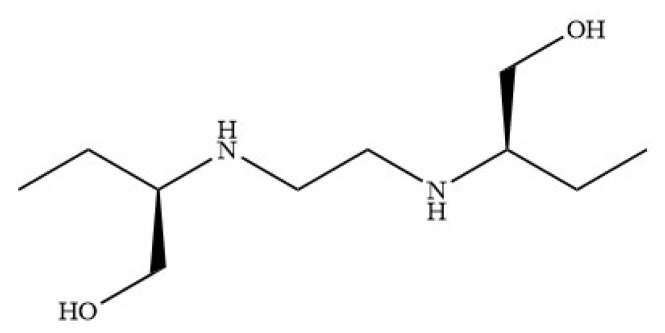
Ethambutol (ETB).

The current available treatments for tuberculosis (TB) infections include a combination of four antituberculosis drugs, namely isoniazid, rifampicin, ethambutol, and pyrazinamide, in the first several months, followed by continued treatment with isoniazid and rifampicin. Ethambutol (ETB) (Figure 2) is bacteriostatic by inhibiting the cell wall synthesis of TB bacteria, and helps to prevent emergence of rifampicin resistance even when resistance to isoniazid is present [7–9]. This conventional long-term treatment is complicated and possesses life threatening adverse effects that lead to patient noncompliance, and further contributes to the development of drug resistant strains. Therefore, improved treatment strategies and drug formulations are deemed necessary to combat the global TB epidemic and the spread of drug resistant TB. Unfortunately, the discovery and development process for newer types of drugs will almost certainly need more than 10 years of continuous expert research and costing. Due to this problem, repurposing existing drugs is a more practical alternative option.

Supramolecules are attractive candidates to be employed into antitubercular drug delivery systems as they are capable to improve the physicochemical properties of desired drugs, mainly on their solubility and bioavailability [10]. These systems aim to locate the drug at specific sites in a controlled manner, in order to reduce the dosage and dosing frequency, with the aim to upgrade therapeutic benefits by increased patient compliance [11]. However, to the best of our knowledge, no previous studies of ETB complexation with β-CD have been described in the literature. There are but some reports with only other antituberculosis drugs, such as isoniazid and rifampicin [12, 13]. Therefore, it would be ideal to propose inclusion complexation using ETB as it may encourage further modification and improvement of this first-line drug.

In the present work, the behaviour of β-CD/ETB inclusion complex was studied to demonstrate its enhanced physicochemical properties, particularly its solubility and thermal properties via UV–visible (UV–Vis) and thermogravimetric analysis (TGA). The preparation (in solid and liquid state) and spectroscopy studies using Fourier transform infrared (FTIR), ^1^H nuclear magnetic resonance (NMR) and nuclear Overhauser effect spectroscopy (NOESY) NMR were successfully presented to elucidate the binding behaviour and molecular interaction between the host and the guest. This fundamental understanding that governs the molecular recognition between β-CD and ETB using spectroscopic methods will provide key insight into the nature of their interaction, which can be extended to improvise the existing drug delivery system today.

## 2. Materials and methods

### 2.1. Chemicals

Beta-cyclodextrin (β-CD) (>97%) was purchased from Sigma-Aldrich Company and used without further purification. Ethambutol (ETB) was obtained from Cayman Chemical Company and used without further purification. Methanol (CH_3_OH) (LC grade), ethanol (C_2_H_5_OH) (95%), and hydrochloric acid (HCl) (37%) were purchased from QRec. Sodium hydroxide (NaOH) (99%) and copper (II) sulphate pentahydrate (CuSO_4_.5H_2_O) were obtained from R&M Chemical. Dimethyl sulfoxide (DMSO-d_6_) (99.8%) was obtained from MagniSolv. Distilled water was used to dilute the solutions.

### 2.2. Preparation of β-CD/ETB complex

The kneading method was used to prepare the β-CD/ETB complex by grinding β-CD and ETB with molar ratio of 1:1. A few drops of ethanol were added to form homogeneous paste. The mixture was ground for 30 min. The sample was then dried and kept in a desiccator to a constant mass.

### 2.3. Characterisation of complex in solid state

The structural information of β-CD, ETB, and β-CD/ETB inclusion complex was studied via various analyses. FT-IR absorption spectra were determined using Perkin-Elmer 2000 spectrometer in the range of 4000 to 400 cm^−1^ via the potassium bromide (KBr) method. To prepare the sample for FT-IR analysis, the desired sample and KBr pellets were ground and mixed in a ratio of 1:3 with a mortar and a pestle. Thermal analysis was performed with TGA by using a Mettler Toledo TGA/SDTA 851E Thermogravimetric Analyzer. The analysis was done over the ambient temperature range of 30 to 900 °C in N_2_ atmosphere to understand the weight loss profile for β-CD, ETB, and β-CD/ETB inclusion complex. A Bruker Avance spectrometer 500 MHz was used to determine the ^1^H NMR and NOESY spectra of β-CD, ETB, and β-CD/ETB inclusion complex by using DMSO-d_6_ as the solvent.

### 2.4. Characterisation of complex in liquid state: determination of formation constant and stoichiometry ratio

Absorption spectra measurements were carried out with Shimadzu UV2600 spectrometer in the range of 200–800 nm. Firstly, the stock solutions of β-CD (0.003 M), ETB (0.01 mM), and CuSO_4_ (0.01 mM) were prepared. Ten millilitres of ETB (0.01 mM) and 10 mL of CuSO_4_ (0.01 mM) were mixed. Next, 2.0 mL of ETB/Cu (0.01 mM) and 3.2 mL of β-CD (0.003 M) solutions were pipetted into a 10-mL volumetric flask to produce the solution of β-CD/ETB/Cu. Distilled water was added to the calibration mark. The absorption spectra for β-CD (0.003M), ETB/Cu (0.01 mM), and β-CD/ETB/Cu were recorded using UV–Vis. The series of β-CD/ETB/Cu solution without pH adjustment (natural) and with pH adjustment (pH 4 and pH 9) were prepared. Each series consisted of fixed concentration of ETB/Cu (0.01 mM) and varied concentration of β-CD (0.002, 0.004, 0.005, and 0.006 M). The Benesi-Hildebrand plot was generated by using Equation (1) [14]. The slope in Equation (2) and coefficient of determination obtained from the plot was applied to determine the formation constant (K) and stoichiometry ratio of inclusion complex formed.


(1) $$\frac{1}{A-A_{0}}=\frac{1}{A^{\prime }-A_{0}}+\frac{1}{K(A^{\prime }-A_{0})[\beta -\text{CD}]}$$

(2) $$K=\frac{1}{Slope(A^{\prime }-A_{0})}$$

## 3. Results and discussions

### 3.1. Characterisation of inclusion complex in solid state: FTIR

**Figure 3:**
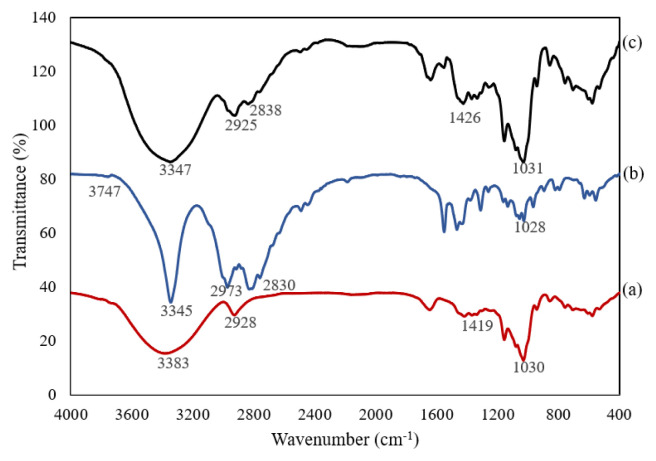
FTIR spectra of a) β-CD, b) ETB, and c) β-CD/ETB.

The FTIR spectra and main frequencies for β-CD, ETB, and β-CD/ETB inclusion complex are reported in Figure 3 and Table 1, respectively. The FTIR spectrum of β-CD (Figure 3a) was characterised by absorption bands at 3383 cm^−1^ (O-H stretching vibration), 2928 cm^−1^ (C-H stretching vibration of methylene group), 1419 cm^−1^ (O-H bending), and 1030 cm^−1^ (C-O stretching vibration of cyclic ether group). ETB (Figure 3b) was characterised by the absorption bands at 3747 cm^−1^ (N-H stretching vibration), 3345 cm^−1^ (O-H stretching vibration), 2973 cm^−1^ and 2830 cm^−1^ (C-H stretching vibrations of methyl and methylene groups), and 1028 cm^−1^ (C-N stretching vibration).

In Figure 3c, the absorption band of the β-CD/ETB inclusion complex was seen to be largely dominated by the vibrational bands of β-CD due to huge molecular size and seven repeating units of D-glucopyranose unit in the β-CD structure [15]. Hence, the FTIR spectrum of the inclusion complex in Figure 3c was almost identical to the FTIR spectrum of β-CD in Figure 3a. Besides, β-CD contained polar functional groups (C-O group and O-H group) which resulted in strong absorption of the vibrational bands [16].

Figure 3c shows the stretching frequencies of N-H stretching of ETB at 3747cm^−1^ had disappeared. The broad O-H stretching band of the inclusion complex at 3347 cm^−1^ corresponded to the multiple O-H functional groups of β-CD molecules as compared to the narrow peak of O-H stretching band from the pure ETB spectrum [17, 18]. Both of C-H absorption bands in ETB (2973 cm^−1^ and 2830 cm^−1^) were found to be overlapped with the C-H band in β-CD (2928 cm^−1^) and had shifted to 2925 cm^−1^ and 2838 cm^−1^ in the inclusion complex. Meanwhile, the C-O cyclic ether group band in β-CD was found to be broadened and intensified (1031 cm^−1^) upon complexation and overlapped with the C-N absorption band in ETB. The FTIR spectrum in the fingerprint region (below 1300 cm^−1^) had confirmed that the β-CD/ETB inclusion complex was different from the parent host and guest molecules, as they possess different spectroscopic signals [19]. These observations of changes in stretching frequencies were because ETB was embedded into the hydrophobic cavity of β-CD, which could be regarded as the evidence of the host–guest interaction between β-CD and ETB.

### 3.2. Thermal analysis

**Figure 4:**
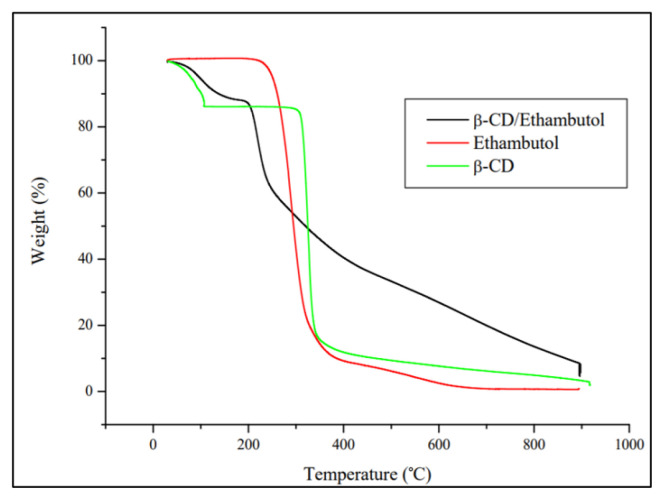
TGA thermogram for β-CD, ETB, and β-CD/ETB.

The thermal stability of ETB, β-CD, and β-CD/ETB inclusion complex was investigated using TGA analysis at the temperature range of 30–900 °C at 20 °C min^−1^. Based on the thermogram shown in Figure 4, pure ETB exhibited a single weight loss at 256–900 °C region. Meanwhile, pure β-CD showed two stages of weight loss. According to Ja’Far et al. (2018), Prabu et al. (2020), and Razak et al. (2014), the first weight loss around 13.75% below 100 °C was due to the loss of water molecules, and another weight loss (84.13%) at 312–900 °C was due to the decomposition of macrocycles [13, 15, 17].

The thermogram of the β-CD/ETB inclusion complex exhibited an initial weight loss of 11.54% at below 100 °C due to loss of moisture, and another weight loss of 80.46% at 205–900 °C. The TGA result showed that β-CD/ETB had lower weight loss than pure ETB and hence exhibited higher thermal stability than pure ETB. The higher stability of the inclusion complex may be due to the strong hydrophobic interaction between β-CD and ETB [20]. However, β-CD/ETB did not undergo complete decomposition, a residual weight of 8% was observed despite reaching 900 °C, this may be caused by the presence of impurities in the sample which may have affected the accuracy of results. Moreover, the percentage of water loss (11.54%) in the inclusion complex was lower than the percentage of water loss (13.75%) in pure β-CD, which indicated that some of the water molecules in the host–guest complex were replaced to allow ETB to enter the β-CD cavity [21]. These results had suggested the molecular interaction between β-CD and ETB. The temperature regions assigned to each weight loss of pure β-CD, ETB, and β-CD/ETB are shown in Table 2.

### 3.3. ^1^H-NMR

Direct convincing proof for the formation of inclusion complex was established using the ^1^H NMR spectroscopy by determining the chemical shifts. This characterisation had provided helpful details on the inclusion mechanism of β-CD host and ETB. The changes in chemical shift for the protons of β-CD and ETB compared to the inclusion complex were defined as induced shift (Δδ). The induced shift was calculated by using Equation (3):


(3) $$\Delta \delta =\delta_{\left(\text{complex}\right)}-\delta_{\left(\text{free}\right)}$$

**Figure 5:**
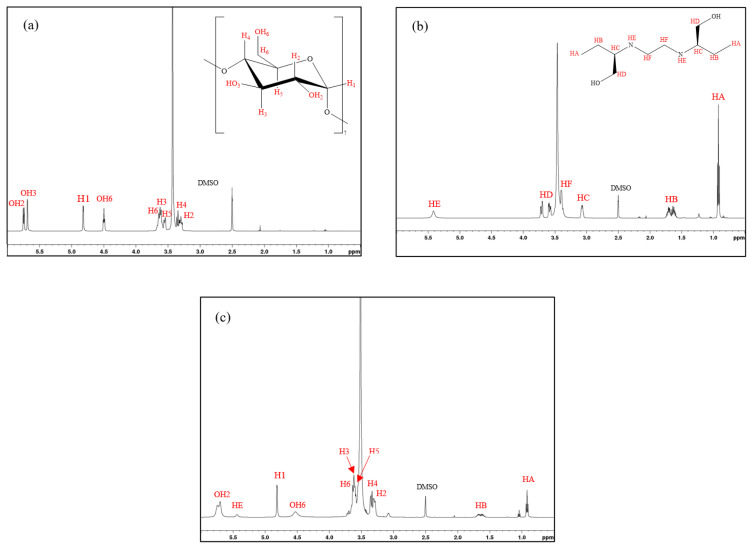
^1^H-NMR spectra of a) β-CD, b) ETB, and c) β-CD/ETB.

The ^1^H NMR spectra and chemical shifts of β-CD, ETB, and β-CD/ETB inclusion complex are shown in Figure 5 and Table 3, respectively. The positive and negative signs of induced shift are denoted as upfield and downfield shifts. The ^1^H NMR spectrum for β-CD comprises six varieties of protons (H1–H6), as illustrated in Figure 5a. Meanwhile, the ^1^H NMR spectrum of ETB contains four varieties of protons (HA-HF), as illustrated in Figure 5b.

**Figure 6:**
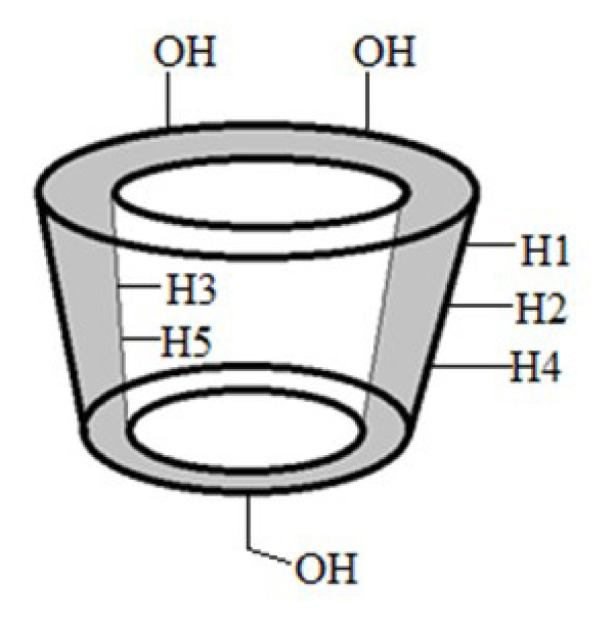
Structure of β-CD.

The structure of β-CD in Figure 6 showed that the protons of H3 and H5 were located inside the hydrophobic cavity, while the other protons (H1, H2, and H4) were located at the hydrophilic exterior. When the inclusion complex is formed, the inner protons (H3 and H5) of the glucose units of β-CD would be affected by the inclusion of the nonpolar region of the guest into the hydrophobic cavity. Hence, the ^1^H NMR spectrum of β-CD/ETB in Figure 5c had shown appreciable Δδ values for H3 and H5 protons, which was –0.011 ppm and 0.044 ppm, as listed in Table 3. The changes of chemical shift for H3 and H5 protons indicated that ETB had penetrated into the hydrophobic cavity of β-CD, due to the magnetic anisotropy effects of ETB [13].

To further investigate the formed inclusion complex, the chemical shifts of ETB compared with the inclusion complex were also examined. The Δδ value of HE proton of ETB, which was 0.020 ppm, was the highest compared to the other protons from the formation of the inclusion complex. This large chemical shift suggested that the HE proton of ETB was located inside the β-CD cavity. Furthermore, the presence of proton signals from both of β-CD and ETB in the ^1^H NMR spectrum (Figure 5c) indicated the formation of an inclusion complex.

### 3.4. NOESY NMR

**Figure 7:**
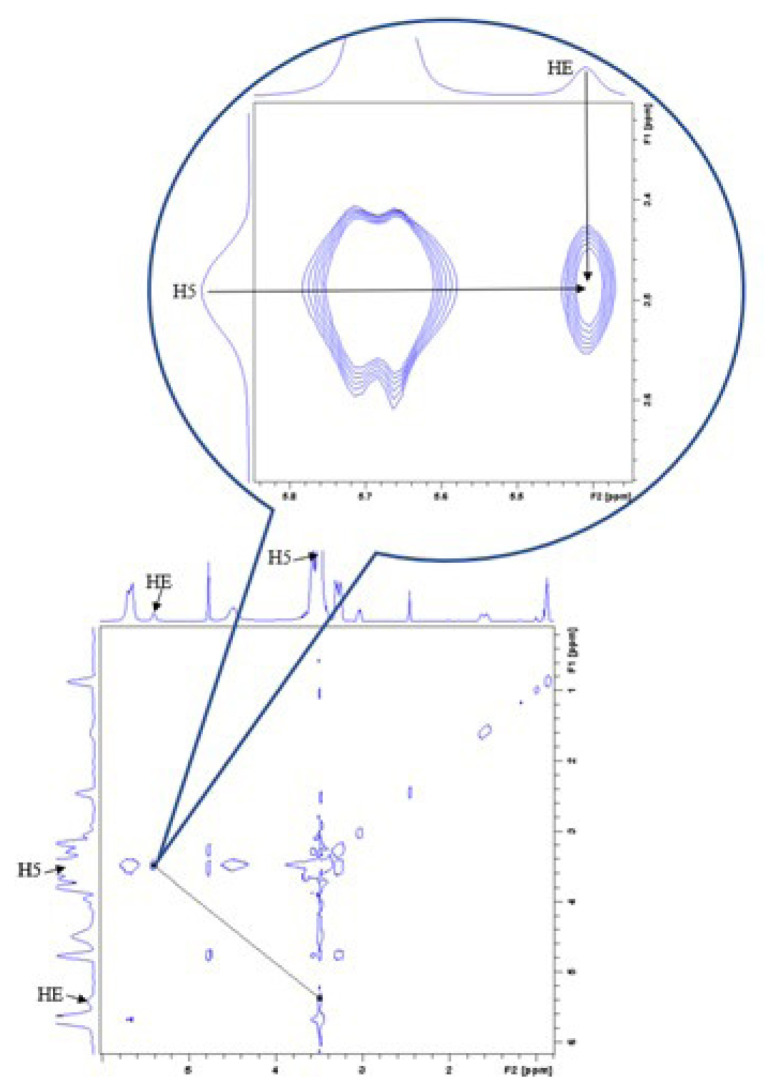
2D NOESY NMR spectra of β-CD/ETB.

Although the placement was not definitive, the possible placement of the ETB in the β-CD cavity was obtained from ^1^H NMR analysis. The ^1^H NMR analysis result was strongly supported by NOESY NMR analysis to examine the encapsulation of ETB into the hydrophobic cavity of β-CD by providing the geometry for the inclusion compound. The analysis provided spatial connection information between neighbouring protons in the cavity of the inclusion complex by observing intermolecular dipolar complex interactions [16, 17]. The cross-peak in Figure 7 was originated from the interaction between protons of β-CD and ETB. The cross peak of the H5 proton from β-CD at 3.42–3.54 ppm and the HE proton from ETB at 5.38–5.43 ppm had demonstrated strong intensity. Therefore, combining the results of ^1^H NMR and NOESY NMR, it can be concluded that amine moiety of ETB had penetrated into the β-CD cavity and inclusion complex was formed through hydrophobic interaction.

### 3.5. Interaction between ETB and β-CD in aqueous medium: UV–Vis

The extent of interaction involved for the inclusion complex formation was determined by observing the changes in UV–Vis absorbance. However, direct ETB determination in UV–Vis range is not feasible due to its low molar absorptivity in aqueous form [8]. Hence, complexation of ETB with Cu(II) was carried out as developed by Faria et al. (2011). Next, the absorbance of ETB/Cu with increasing concentration of β-CD was measured. The stoichiometric ratios and the formation constants in an aqueous medium were also determined.

**Figure 8:**
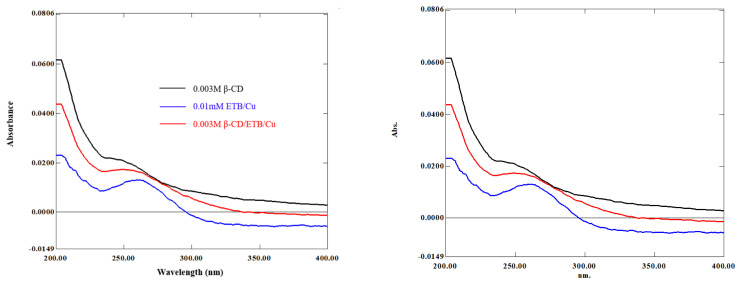
Absorption spectra of β-CD, ETB/Cu, and β-CD/ETB inclusion complex.

The absorption spectra of β-CD (0.003 M), ETB/Cu (0.01 mM), and inclusion complex were shown in Figure 8. The absorption band of ETB/Cu had obtained a maximum peak at 257 nm, meanwhile, β-CD showed little to no absorbance due to the absence of π electrons or nonbonding electrons [16, 17]. Upon the addition of ETB/Cu into β-CD, the absorption band of the complex had slightly blue-shifted to a shorter wavelength at 251 nm, which indicated the encapsulation of ETB/Cu into the β-CD cavity. It was observed that the absorbance of the complex underwent the hyperchromic effect, where the intensity of absorbance peak was increased. The hyperchromic effect was due to the presence of hydrogen bonding that lowered the energy of ‘n’ orbitals [16]. The absorbance of the guest drug will be enhanced upon complexation with the β-CD cavity because the excited species from nonradiative processes occurring in the bulk solution was shielded and the molar absorption coefficient of the inclusion complex was also increased [17].

**Figure 9:**
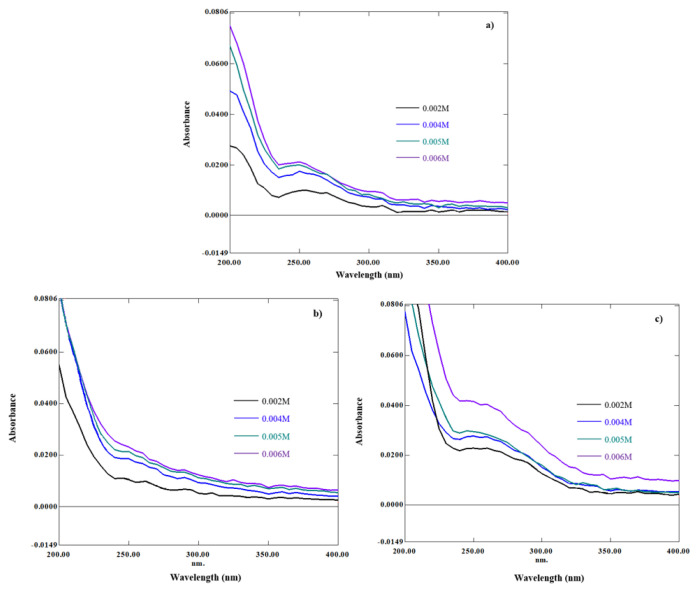
Absorption spectra of ETB/Cu with increasing concentration of β-CD at a) Natural pH, b) pH 4, and c) pH 9.

In Figure 9, the absorption spectra of ETB/Cu were increased with increasing concentrations of β-CD (0.002 M, 0.004 M, 0.005 M, 0.006 M) at natural pH (pH 5.65), pH 4, and pH 9, due to the increasing molar absorptivity of ETB. The UV–Vis analysis was carried out at three different pH values to find out whether the binding strength of ETB to the β-CD cavity will be affected in acidic or basic condition.

**Figure 10:**
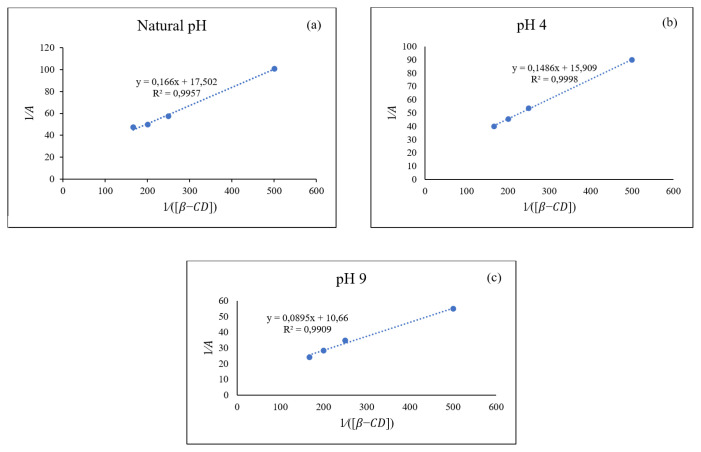
Reciprocal plots of against at a) Natural pH, b) pH 4, and c) pH 9.

The formation constant, K value, and stoichiometric ratio of β-CD and ETB were determined using the Benesi-Hildebrand equation [22]. The reciprocal plot in Figure 10 had clearly indicated that the stoichiometry ratio of the formation of inclusion complex between ETB and β-CD was 1:1, because a good linearity with correlation coefficient of R^2^ > 0.99 was obtained. The K values at various conditions such as natural pH (pH 5.65), acidic (pH 4), and basic (pH 9) were calculated using Equation (2) to determine the binding strength of molecules in the complex of ETB/Cu and β-CD. The K values of complexes are shown in Table 4. It was found that the difference in K values for the complexes in natural, acidic, and alkaline conditions were insignificant. Hence, the natural condition of the inclusion complex could be chosen as the optimum pH due to the ease of preparation.

## 4. Conclusion

In conclusion, the formation of β-CD/ETB inclusion complex prepared with the kneading method was confirmed in the present study. The FTIR analysis had verified the encapsulation of ETB into the hydrophobic cavity of β-CD, while the inclusion complex had lower weight loss than pure ETB and hence exhibited higher thermal stability than pure ETB, which was proven in the TGA results. According to the induced shifts of the inclusion complex in ^1^H NMR, large shifts were observed for the H3 and H5 protons from β-CD and the HE proton from ETB, which indicated that ETB had penetrated into the hydrophobic cavity of β-CD. The result of ^1^H NMR was further proven by the NOESY NMR result, where the cross peak of H5 proton from β-CD and the HE proton from ETB were found. Thus, it was concluded that ETB had penetrated into the cavity of β-CD and an inclusion complex was formed through hydrophobic interaction. Moreover, the stoichiometry ratio of β-CD:ETB was proven to be 1:1 by using the Benesi-Hildebrand equation. UV–Vis analysis was carried out by using three different pH values and the K values for natural pH, pH 4, and pH 9 were 105.43, 107.06, and 119.11, respectively, which had insignificant differences. To sum up, this integrated experimental method had produced a β-CD/ETB inclusion complex that could be further investigated for future application in the drug carrier system.

## Figures and Tables

**Table 1:** Main FTIR frequencies for β-CD, ETB, and β-CD/ETB.

Wavenumber, cm^−1^	Functional group	β-CD	ETB	β-CD/ETB
3747	N-H stretching (Secondary amine)	-	√	-
3383	O-H stretching (Alcohol)	√	-	-
3347		-	-	√
3345		-	√	-
2973	C-H stretching (Methyl and methylene group)	-	√	**-**
2928		√	-	-
2925		-	-	√
2838		-	-	√
2830		-	√	-
1426	O-H bending	-	-	√
1419		√	-	-
1031	C-O stretching (Cyclic ether)	-	-	√
1030		√	-	-
1028	C-N stretching	-	√	-

**Table 2:** Temperature of weight loss for β-CD, ETB, and β-CD/ETB.

Samples	Region, °C	Weight Loss, %	Assignment
β-CD	<100	13.75	Loss of moisture
	312–900	84.13	β-CD decomposition
ETB	256–900	99.19	ETB decomposition
β-CD/ETB	<100	11.54	Loss of moisture
	205–900	80.46	β-CD and ETB decomposition

**Table 3:** Chemical shifts corresponding to β-CD, ETB, and β-CD/ETB.

Proton	β-CD, δ	ETB, δ	Inclusion complex, δ	Induced shift, Δδ
H1	4.817	-	4.810	−0.007
H2	3.297	-	3.305	0.008
H3	3.626	-	3.615	−**0.011**
H4	3.361	-	3.356	−0.005
H5	3.541	-	3.585	**0.044**
H6	3.640	-	3.634	−0.006
HA	-	0.916	0.917	0.001
HB	-	1.635	1.639	0.004
HC	-	3.075	3.082	0.007
HD	-	3.701	-	-
HE	-	5.416	5.436	**0.020**
HF	-	3.402	-	-

* Bold values refer to the highest induced shift for that particular proton.

**Table 4:** Formation constant (K) values for β-CD/ETB at various conditions.

pH	Formation constant, K
Natural	105.43
4	107.06
9	119.11
